# The neuro-immune axis in cancer: mechanisms of innervation-driven tumor progression and therapeutic opportunities

**DOI:** 10.3389/fimmu.2025.1693419

**Published:** 2026-01-12

**Authors:** Liang Zhang, Dantong Zhu, Junyi Wang, Jiyang Guo, Mingzhe Jiang, Xinhui Qi, Zhuo Zhang, Zhendong Zheng

**Affiliations:** 1 Department of Oncology, General Hospital of Northern Theater Command, Shenyang, Liaoning, China; 2 Department of Human Anatomy, College of Basic Medical Sciences, China Medical University, Shenyang, Liaoning, China

**Keywords:** angiogenesis, cancer-associated axons, innervation, therapeutic strategies, tumor biology, tumor microenvironment

## Abstract

Innervation plays a key role in tumor progression, and malignant tumor cells often invade peripheral nerve or the central nervous system, significantly altering tumor biological function. In this review, the multi-dimensional role of innervation in cancer biology is comprehensively discussed, the molecular mechanisms involved and their effects on the tumor microenvironment are deeply analyzed, and therapeutic strategies are proposed. We systematically summarize the interactions between cancer cells and neural tissue, focusing on how key signaling pathways regulate the core elements of this process. The analysis focused on the pathological features of innervation in specific cancer types, particularly breast, pancreatic, and prostate cancers, revealing the unique mode of action of innervation in these cancers. In addition, we explored the combined effects of innervation on the tumor microenvironment, including immune cell infiltration, angiogenesis, metabolic reprogramming, and the development of cancer-related pain. Together, these changes promote tumor growth and spread, further highlighting the importance of innervation in tumor progression. Finally, this review proposes the potential therapeutic value of innervation in the treatment of cancer, and aims to promote the development of the field of innervation research. A deeper understanding of the complex relationship between innervation and cancer progression is critical to optimizing treatment strategies, improving patient outcomes, and expanding the boundaries of our understanding of cancer biology.

## 1 Introduction

Cancer has emerged as a pressing health concern on a global scale, acknowledging widespread recognition of its profound and far-reaching impacts across various sectors. (1). In 2022, 23.56 million new cancer cases were diagnosed worldwide, resulting in approximately 17 million deaths, highlighting the magnitude of this public health problem (2). Cancer is characterized by the uncontrolled proliferation of malignant cells caused by genetic mutations, often accompanied by aggressive and metastatic behavior (3). Notably, there are significant differences in regulatory mechanisms between primary and metastatic tumors, underscoring the importance of delving deeper into their different biological behaviors (4). The pathological pathogenesis of cancer is extremely complex, not only dynamic in time, but also significantly heterogeneous in space, which poses a severe challenge to the formulation of treatment strategies and requires a comprehensive approach to clinical decision-making (5). Despite many positive advances in oncology research, traditional treatments such as chemotherapy, radiation, and surgery still have limitations, including toxic effects, potential damage to nearby healthy tissue, and high economic burdens (6). These challenges span all stages of cancer diagnosis through clinical management, leading to unmet patient needs and significant treatment gaps.

In view of this, identifying functional biomarkers is critical to improve early cancer detection and develop effective long-term treatment strategies (7). In addition, in-depth understanding of the molecular mechanisms that drive cancer progression is also of great significance for optimizing existing therapies and improving patient outcomes. Therefore, this review aims to comprehensively analyze the latest advances in cancer research and provide theoretical support and direction for future clinical practice. The nervous system plays a vital role in the modulation of cancer initiation and has a profound impact on tumor progression and dissemination (8). Notably, cancer cells can remodel the nervous system’s architecture and functionality to promote their proliferation, as evidenced by studies demonstrating tumor-induced neurogenesis and axonogenesis in preclinical models (9).Accumulating evidence links tumor innervation to cancer prognosis: for instance, in pancreatic ductal adenocarcinoma (PDAC), increased perineural invasion correlates with enhanced metastatic potential and reduced overall survival (10). Similarly, in prostate cancer, nerve density within tumors independently predicts biochemical recurrence (11). These findings collectively suggest that tumor nerve density may serve as a pan-cancer prognostic biomarker, though further validation across diverse malignancies is warranted.

An elevated concentration of nerve fibers within tumors has been correlated with diminished survival rates, an increased likelihood of metastasis, and a heightened risk of recurrence in various solid tumors, notably pancreatic and prostate cancers. This association implies that nerve density may function as a valuable prognostic indicator in oncological practice. Tumor cells leverage their interactions with the nervous system by releasing neurotrophic factors that facilitate nerve proliferation within the tumor microenvironment (12). Subsequently, these activated nerves can secrete growth factors that not only stimulate tumor progression but also assist in metastatic processes (13). The complex interplay between tumor innervation and cancer biology represents a significant domain for investigation. Gaining insights into this relationship could lead to the development of innovative therapeutic approaches aimed at disrupting these pathways, ultimately enhancing patient prognoses.

In essence, tumor innervation’s pivotal role in cancer progression, modulating the TME and patient outcomes, necessitates a deep understanding of its mechanisms and targeted therapies. This review encapsulates tumor innervation’s intricacies, from molecular mechanisms to TME impacts and therapeutic exploitation of the nervous innervation in cancer management, underscoring its significance in cancer research and clinical practice.

## 2 Cancer–nerve regulation

### 2.1 Nerves and tumorigenesis

Nerves play a crucial role in the cellular microenvironment of humans, serving as conduits that connect various body regions to the central nervous system. They have a profound impact on motor functionalities and the regulation of internal organs. Within the tumor microenvironment, cancer cells can induce nerve growth by secreting neurotrophic factors, which not only promote nerve development but also aid in cancer advancement. Nerves have been perceived as passive structures that cancer cells merely encroach upon, a phenomenon referred to as perineural invasion. However, groundbreaking research conducted over the past five years has revealed the critical role of innervation in tumors. These investigations suggest that nerves actively participate in both the initiation and progression of cancer, thereby challenging the conventional perspective and underscoring their significance in the cancer development process in **Table 1**.

**Table 1:** Key Roles of Nerve Innervation in Tumor Initiation and Progression.

Cancer type	Reference	Functions/unsolved problems
Colorectal cancer	PMID: 39137067 Neuro-Mesenchymal Interaction Mediated by a β2-Adrenergic Nerve Growth Factor Feedforward Loop Promotes Colorectal Cancer Progression	**Functions:** Identifies a feedforward loop where norepinephrine induces CAFs to secrete NGF, increasing sympathetic innervation and promoting tumor growth via ADRA2A/YAP and NGF/Trk/AKT pathways. **Unsolved:** Clinical efficacy and potential side effects of targeting this neuro-mesenchymal interaction with TRK inhibitors in patients remain unexplored.
Gastric cancer	PMID: 25367956 Vagal innervation is necessary for gastric tumorigenesis	**Functions:** Provides evidence that the vagus nerve is required for gastric stem cell expansion and gastric tumorigenesis. **Unsolved:** The applicability, specificity, and long-term consequences of vagal denervation as an anti-tumor strategy in humans are unresolved.
Prostate	PMID: 19047084 Cancer-related axonogenesis and neurogenesis in prostate cancer	**Functions:** This study reveals that prostate cancer tumors can induce and are innervated by newly formed nerves, a process termed cancer-related axonogenesis and neurogenesis, which contributes to tumor growth and progression. **Unsolved:** Key unsolved problems include the precise cellular origins of the new nerves, the complete signaling pathways driving this neurogenesis, and how this process can be therapeutically targeted.
	PMID: 19509179 Global gene expression analysis of reactive stroma in prostate cancer	**Functions:** The study defines a reactive stroma gene expression signature in human prostate cancer, identifying stromal cell differentiation, angiogenesis, and mitogen signaling as key pathways associated with progressive disease. **Unsolved:** Key unsolved problems include the elucidation of the specific molecular triggers that initiate the reactive stroma program and the development of strategies to therapeutically target this pro-tumorigenic microenvironment.
Breast	PMID: 39232464 Global and single-cell proteomics view of the co-evolution between neural progenitors and breast cancer cells in a co-culture model	**Functions:** Shows stromal DCX+ neural progenitor cells are associated with aggressive breast cancer and poor survival; proteomics reveals co-evolution changes. **Unsolved:** The specific functional mechanisms through which neural progenitors influence breast cancer progression remain to be elucidated.
Pancreatic	PMID: 41005304 Sensory neurons drive pancreatic cancer progression through glutamatergic neuron-cancer pseudo-synapses	**Functions:** Identifies functional pseudo-synapses between sensory neurons and PDAC cells, with glutamate signaling through GRIN2D receptors promoting progression. **Unsolved:** The clinical relevance and specific strategies for targeting neuron-cancer pseudo-synapses in human patients are currently unknown.
	PMID: 33142117 Neurons Release Serine to Support mRNA Translation in Pancreatic Cancer	**Functions:** Reveals metabolic crosstalk where neurons provide serine to PDAC cells under nutrient stress, supporting growth, while cancer cells secrete NGF to promote innervation. **Unsolved:** The full scope of nutrient exchange and whether other neuron-derived metabolites contribute to tumor survival is not fully understood.
	PMID: 28386018 PanIN Neuroendocrine Cells Promote Tumorigenesis via Neuronal Cross-talk	**Functions:** Identifies neuroendocrine PanIN cells that respond to sensory neuron-derived Substance P via NK1-R/STAT3 signaling to drive proliferation. **Unsolved:** The clinical exploration of targeting neuroepithelial cross-talk (e.g., NK1-R inhibition) in PDAC prevention or treatment is needed.
Head and neck	PMID: 38423011 Sensory nerve release of CGRP increases tumor growth in HNSCC by suppressing TILs	**Functions:** This study demonstrates that sensory nerves promote HNSCC progression by releasing the neuropeptide CGRP, which directly suppresses the anti-tumor function of tumor-infiltrating lymphocytes (TILs) within the tumor microenvironment. **Unsolved:** Key unsolved problems include identifying the specific immune cell subsets and intracellular signaling pathways targeted by CGRP, and determining whether blocking this neuro-immune axis can be translated into an effective therapeutic strategy for patients.
	PMID: 32051587 Loss of p53 drives neuron reprogramming in head and neck cancer	**Functions:** The study demonstrates that loss of p53 promotes neuron reprogramming in head and neck cancer cells, potentially enhancing tumor plasticity and progression. **Unsolved:** The research may leave unresolved questions regarding the precise molecular mechanisms of neuron reprogramming and its therapeutic targeting in clinical settings.
	PMID: 41138728 Cancer cells co-opt an inter-organ neuroimmune circuit to escape immune surveillance	**Functions:** Discovers that cancer-derived SLIT2 activates nociceptive neurons, leading to CGRP-mediated immunosuppression in TDLNs and facilitating immune escape. **Unsolved:** The broader applicability of this inter-organ neuroimmune circuit across other cancer types and the optimal therapeutic strategies for its disruption need investigation.

Functions: The issues that have been addressed in the references related to axonogenesis and tumor development.

Unsolved: The issues that remain unsolved in the references related to axonogenesis and tumor development.

A substantial body of research has underscored the pivotal role of nerves in cancer initiation and progression. (14) demonstrated that autonomic nerve development within the tumor microenvironment is a key driver of prostate cancer progression, with sympathetic nerves promoting early tumorigenesis and parasympathetic nerves facilitating metastatic dissemination. Mechanistically, tumor cells secrete neurotrophic factors such as nerve growth factor, brain-derived neurotrophic factor, and glial cell-derived neurotrophic factor and release axon-guidance molecules such as ephrin B1 to promote axonogenesis (15). In prostate cancer, this phenomenon correlates with increased neuronal density in peritumoral ganglia, which is associated with a 40% higher risk of biochemical recurrence (16). Critically, this tumor-specific neurogenic program differs from the regenerative mechanisms in healthy tissues: while injured sympathetic nerves in the normal prostate regenerate via axonal sprouting and synaptic remodeling (17), cancer-associated neurogenesis involves *de novo* formation of neuronal cells, highlighting a distinct pathological pathway.

The research findings indicate that using denervation strategies in mouse models of prostate cancer can significantly slow down the progression of the disease (18). This discovery underscores the role of the sympathetic nervous system, particularly the adrenergic system, and the parasympathetic nervous system, known as the cholinergic system, in regulating the physiological functions of the prostate (19). Additionally, the study shows that denervating the prostate, whether through surgical or chemical methods, can greatly diminish the growth of prostate cancer cells and their dissemination throughout the body.

In cases of prostate cancer, newly formed autonomic nerves infiltrate the tumor microenvironment. This infiltration facilitates cancer development and progression by activating β-adrenergic and cholinergic signaling pathways. The identification of doublecortin (DCX)-positive cells within the stroma of primary human prostate cancers, which also express markers such as PSA-NCAM and Internexin, reinforces this assertion (20). Mauffrey et al. further elucidate that neural progenitors, originating from the subventricular zone, can migrate to sites of cancer and metastasis, where they differentiate into adrenergic phenotypes. Among prostate cancer patients stratified into high- and low-risk categories, the density of DCX-positive cells exhibits a strong correlation with the aggressiveness of the cancer and the probability of recurrence, thereby highlighting their involvement in initiating neurogenesis and facilitating malignant progression (21).

Cancer cells do not directly transdifferentiate into mature neurons but instead create a neurogenic microenvironment through cancer stem cell (CSC)-mediated paracrine signaling (22). Emerging evidence demonstrates that CSCs isolated from gastric and colorectal cancers secrete neurotrophic factors such as nerve growth factor (NGF) and brain-derived neurotrophic factor (BDNF), which promote axonal growth and sympathetic/cholinergic marker expression in peripheral neurons. For instance, *in vitro* co-culture experiments revealed that gastric CSC-conditioned medium induced tyrosine hydroxylase (TH) expression in sympathetic neurons and vesicular acetylcholine transporter (VaChT) expression in cholinergic neurons, without evidence of CSC-to-neuron transdifferentiation (23). These findings align with established evidence indicating that neurotrophins (e.g., NGF, BDNF) regulate cancer stem cell (CSC) stemness by promoting their proliferation, survival, and enrichment while maintaining stemness markers (e.g., SOX2, OCT4) under microenvironmental (24). Thus, the neurogenic capacity of cancers primarily relies on CSC-driven remodeling of the peripheral nervous system rather than direct neuronal differentiation.

### 2.2 Mechanistic insights into neural modulation of tumorigenesis

**Figure 1:**
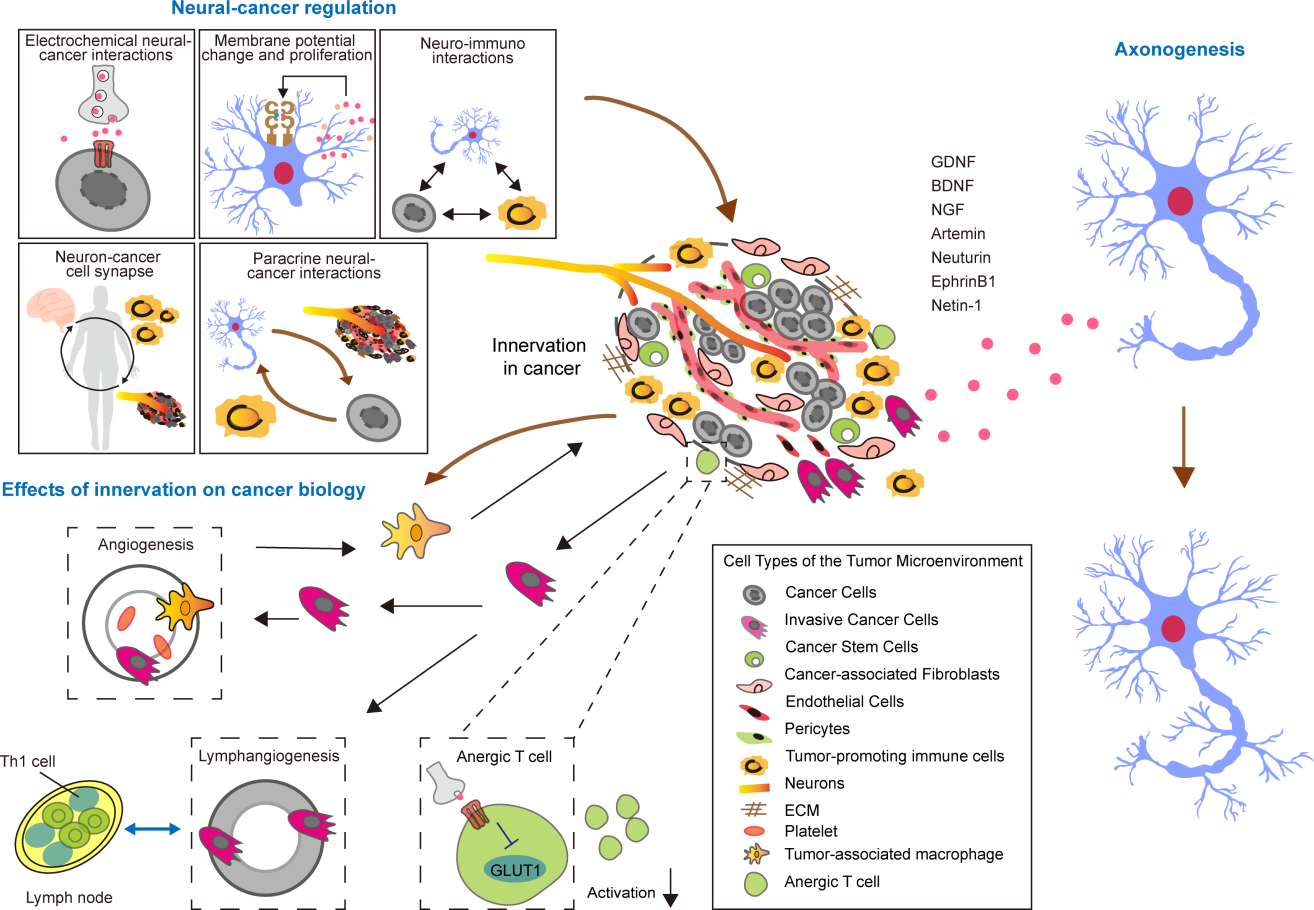
Tumor-mediated neurogenesis, neuro-tumor interactions, and the influence of innervation on tumor biology in TME.

The interplay between neural structures and tumors is intricate, encompassing multiple interconnected facets. These include direct communication through electrochemical signaling, modulation via paracrine signaling, synaptic connections between neurons and cancer cells, changes in membrane potential that could impact cancer cell proliferation, and the interactions of neurotransmitters with the immune system. Together, these various mechanisms create a sophisticated network that links the nervous system to cancer (**Figure 1**).

In terms of direct electrochemical communication, both neurons and cancer cells have the capacity to exchange information through electrochemical signals. This interaction occurs when neurotransmitters released by neurons bind to specific receptors on the surface of cancer cells (25). An example of this is seen with neurotransmitters such as glutamate, which can engage with particular receptors on cancer cells, consequently influencing their functions and growth patterns (26).

Neurons and cancer cells engage in a complex interaction through multifaceted communication mechanisms. This includes paracrine signaling, where signaling molecules such as neurotransmitters and growth factors released by neurons can significantly impact cancer cells, affecting their growth, migration, and invasion capabilities. Remarkably, recent evidence reveals an even more direct form of support, where sensory neurons can donate functional mitochondria to cancer cells via extracellular vesicles, thereby enhancing the metastatic capacity of the receiving tumor cells (27).In turn, cancer cells can secrete specific factors that modulate neuronal activity and synaptic plasticity, highlighting the intricate relationship between the nervous system and cancer. Neurotrophic factors, including glial cell line-derived neurotrophic factor (GDNF), brain-derived neurotrophic factor (BDNF), nerve growth factor (NGF), Artemin, Neurturin, EphrinB1, and Netrin-1, play a vital role in stimulating the proliferation of neural fibers by interacting with specific receptors on neurons, thus influencing neural fiber formation (28, 29). Additionally, tumor cells can recruit neural progenitor cells via the circulatory system, where these progenitor cells are influenced by components of the tumor microenvironment (TME) and can differentiate into fully functional neuronss (30). Moreover, these neurons can directly regulate cancer stem cells (CSCs) through a variety of signaling pathways, further complicating the interplay between the nervous system and cancer (**Figure 1**).

Neurons and cancer cells possess the capacity to establish genuine synaptic links, enabling neurons to directly affect the biological activities of cancer cells through electrochemical signaling. A prominent illustration of this interaction is evident in glioma tumors, wherein glutamatergic synapses may form between neurons and glioma cells, and this phenomenon is not restricted to gliomas. Recent research demonstrates that functional, excitatory glutamatergic synapses also form between neurons and small cell lung cancer cells, which similarly promote tumor proliferation through postsynaptic depolarization, indicating a broader oncogenic role for neuro-synaptic signaling across cancer types (31). These synaptic connections are instrumental in facilitating the proliferation and invasion of glioma tumors, underscoring the substantial influence that neuronal signaling has on the advancement of cancer (32). Synaptic connections function via a unidirectional transmission pathway, wherein signals propagate from presynaptic neurons to postsynaptic neuroglial cells. This process instigates excitatory postsynaptic currents (EPSCs), which are predominantly modulated by calcium-permeable AMPA receptors (AMPAR), leading to the depolarization of the postsynaptic cell (32). Moreover, the direct depolarization of neuroglial cells through optogenetic techniques further stimulates their proliferation. Conversely, indirect perisynaptic contacts exhibit characteristics typical of astrocytes within tripartite synapses, a phenomenon observed in the context of breast cancer brain metastases and adult glioblastoma. In the case of breast cancer brain metastases, these perisynaptic formations are instrumental in facilitating tumor expansion by transmitting glutamatergic signals to NMDA receptors present on the membranes of breast cancer cells (33).Alterations in membrane potential are associated with the proliferation of cancer cells, and fluctuations in neuronal membrane potential can also impact this process. In the case of gliomas, the depolarization of the membrane in cancer cells may act as a catalyst for the growth of gliomas (34). This change in membrane potential could potentially promote the proliferation of glioma cells through pathways that depend on voltage fluctuations.

Neurotransmitters are crucial modulators of immune system activity, which consequently can influence the advancement of cancer. For instance, neurotransmitters such as norepinephrine may stimulate the proliferation and maturation of immune cells, thereby augmenting their capacity to combat cancerous cells. In contrast, neurotransmitters like γ-aminobutyric acid (GABA) can inhibit the functions of immune cells (35). The differential impacts of these neurotransmitters on immune responses can lead to alterations in the immune microenvironment that encircles tumors, thereby affecting the intricate equilibrium between the body’s anti-tumor immune mechanisms and the inflammatory processes that could potentially facilitate cancer progression.

### 2.3 Denervation as a strategy to inhibit tumor neural signaling

Investigations utilizing murine models have demonstrated that denervation significantly impedes the advancement of prostate cancer (36). Both surgical and chemical denervation of the prostate markedly restrict the proliferation and dissemination of prostate malignancies (37). In the initial phases of tumorigenesis, the ablation of adrenergic nerves or the genetic knockout of adrenergic receptors β2 (ADRβ2) and β3 (ADRβ3) leads to a decrease in cancer cell proliferation (38). In contrast, during the later stages of cancer progression, the denervation of parasympathetic nerves or the inhibition of muscarinic acetylcholine receptor 1 (CHRM1) obstructs the metastasis of tumor cells (39). Importantly, the density of neural infiltration, which serves as a marker for axonal growth, is notably elevated in advanced prostate cancer compared to both early-stage malignancies and benign prostatic hyperplasia (40). This finding suggests that adrenergic signaling, mediated by catecholamines released from sympathetic nerves, enhances tumor growth, whereas cholinergic signaling, stimulated by parasympathetic nerves, aids in tumor metastasis.

Recent advancements in the field of prostate cancer research have motivated scientists to explore similar investigations across different cancer types. For instance, research utilizing murine models of gastric cancer indicates that severing the vagus nerve can impede the progression and growth of tumors. Additionally, the inhibition or genetic ablation of the muscarinic acetylcholine receptor 3 (CHRM3) yields effects akin to those observed with vagal denervation, highlighting its significant influence on tumor dynamics (41, 42). These findings imply that targeting neural signaling pathways may pave the way for novel therapeutic strategies in cancer treatment.

In summary, the complex interplay between neuronal cells and cancer highlights the critical need for a more profound comprehension of the mechanisms involved. Nerves contribute to cancer development in several ways; they not only aid in tumor initiation and enhance metastatic potential but also engage with cancer stem cells, illustrating their diverse functions in tumor progression. Consequently, approaches aimed at interrupting these nerve-cancer interactions, such as the excision of nerves and the inhibition of specific receptors, present potential avenues for innovative cancer therapies.

## 3 Cancer-associated axonogenesis: an emerging hallmark of cancer

### 3.1 Characteristics of cancer-associated axons

In the field of cancer biology, the significance of nerves and their axonal structures is frequently overlooked, resulting in a limited comprehension of the interplay between neuronal growth and the tumor microenvironment (TME) (43). Conventional histological assessments predominantly concentrate on larger nerve trunks to evaluate perineural invasion (44). Nonetheless, the functions of smaller nerve fascicles and individual axons within the TME remain inadequately defined. This deficiency highlights the necessity for advanced immunohistochemical (IHC) methodologies that specifically identify neuronal biomarkers (45). Rectifying this neglect is essential. A comprehensive exploration of cancer-related axonogenesis may provide critical insights into tumor development and pave the way for novel therapeutic strategies (46).

Axons linked to cancer display unique characteristics that differentiate them from those found in non-cancerous tissues (47). The complex arrangement of fine nerve fibers and individual axons within the tumor microenvironment (TME) presents a considerable obstacle for conventional histological examinations, as these fragile structures are frequently neglected (48). Although larger nerve bundles are readily identifiable, the intricate microscopic architecture of neuronal infiltration within tumors is often obscured by the TME’s complexity (43). Consequently, there is a pressing need for the application of immunohistochemical (IHC) staining methods that specifically target neuronal markers. Such techniques facilitate the visualization and quantification of these minute structures (49). The presence of numerous small nerve fascicles and individual axons underscores the dynamic process of neuronal remodeling that occurs in response to tumor progression.

### 3.2 Discovery of cancer-associated axonogenesis

Recent advancements in co-culture methodologies have uncovered a novel and active interplay between cancer cells and neurons. Investigations have demonstrated that dorsal root ganglia (DRG) from mice extend projections towards prostate cancer cells (50). This interaction initiates a reciprocal differentiation process, resulting in phenotypic alterations in both neural and cancerous cells (51). Critically, this relationship is not merely structural but functionally pro-tumorigenic. Sensory neurons, for example, enhance the invasion and metastasis of triple-negative breast cancer cells by secreting the axon guidance molecule PlexinB3, which directly activates a pro-metastatic signaling pathway within the cancer cells (52). Notably, this bi-directional communication is marked by a decrease in the spatial separation between cancer and neural cells, coupled with enhanced intercellular signaling, thereby underscoring the formation of an active cancer-nerve communication network that fuels disease progression. The presence of axonal structures across various organs and tissues contributes to an increased density of neural cells, which represents a pivotal feature of the tumor microenvironment (TME).

### 3.3 Cancer-specific features of axonogenesis

Investigations into the development of nerve fibers associated with various tumor types have yielded significant insights. Ayala et al. undertook pioneering studies focused on prostate cancer, employing both two-dimensional and three-dimensional reconstructions. Their results revealed a marked enhancement in both the density and size of neural structures within patient tissues, thereby substantiating the presence of axonogenesis in relation to cancer (53). Notably, axonogenesis has also been observed in precancerous lesions, suggesting its potential role as an early event or as a contributing factor in the advancement of prostate cancer (54).

At the core of this phenomenon are semaphorins, an extensive family of molecules that play a pivotal role in directing axonal growth, with semaphorin 4F (Sema4F) identified as a key player (55). Sema4F, a transmembrane semaphorin, exerts its biological functions primarily by binding to its high-affinity receptor complex. The canonical receptor for many class 4 semaphorins, including Sema4F, is neuropilin-2 (NP2), often in conjunction with a plexin coreceptor (56). This binding is mediated through the Sema domain of Sema4F interacting with specific domains on NP, such as the a1/a2 and b1/b2 domains (57). The engagement of Sema4F with the NP receptor complex is a critical step for initiating downstream signaling cascades that regulate cytoskeletal dynamics and neurite outgrowth (58).

The binding of Sema4F to the NP-Plexin receptor complex triggers a well-characterized intracellular signaling pathway. The primary downstream effectors are the small GTPases of the Rho family. Upon receptor activation, the intracellular domain of plexin interacts with and activates specific Rho GTPase regulatory proteins. This leads to the inactivation of Rac1 and Cdc42, which are promoters of actin polymerization and membrane protrusion, while concurrently activating RhoA, which stimulates actomyosin contractility (59). Investigations have demonstrated that the overexpression of Sema4F in DU-145 prostate cancer cells enhances neurite outgrowth when these cells are co-cultured with PC-12 neuronal cells (60). This enhancement is likely attributed to interactions involving neuropilin (NP) PDZ-binding motifs, which facilitate the localization and assembly of the signaling complex (61). The precise modulation of this Rac/Rho balance results in the reorganization of the actin and microtubule cytoskeleton, culminating in the guidance of growth cones and the regulation of neurite extension.

Importantly, the axon guidance system involves significant cross-talk between different ligand-receptor families. Beyond the NP-Plexin axis, Sema4F can also engage in functional interactions with Roundabout (Robo) receptors, which are classical receptors for Slit proteins. This interaction creates a complex regulatory network where guidance cues are integrated. For instance, studies have shown that Robo receptors can modulate the signaling output of Semaphorins by forming heteromeric complexes with plexins or by competing for shared downstream effectors. This Robo-Semaphorin interplay allows for fine-tuning of neuronal navigation and may explain context-dependent outcomes of Sema4F signaling (62).

Furthermore, the activity of Sema4F is intricately linked with neurotrophic factor signaling pathways. Neurotrophic factors, such as Nerve Growth Factor (NGF) and Brain-Derived Neurotrophic Factor (BDNF), primarily signal through Trk receptor tyrosine kinases to promote neuronal survival and growth. Evidence suggests that Semaphorin signaling can converge with or modulate Trk receptor signaling. For example, Sema4F-induced cytoskeletal remodeling may prime the cellular response to neurotrophins, and conversely, neurotrophin signaling can regulate the expression or localization of semaphorin receptors (63). This bidirectional crosstalk indicates that Sema4F does not act in isolation but is part of a coordinated signaling network where guidance cues and survival/growth factors synergistically or antagonistically regulate neuronal morphology and connectivity.

In comparison to the control group, Sema4F resulted in an approximate threefold increase in neurite length. Furthermore, the application of small interfering RNA (siRNA) inhibitors successfully mitigated this excessive neurite elongation (64). This notable discovery indicates that cancer cells possess the ability to secrete neurotrophic factors and guidance molecules like Sema4F that facilitate axonal development, thereby revealing a novel aspect of the interplay between cancerous and neuronal cells. The mechanistic dissection of Sema4F signaling—through NP binding, Rho GTPase regulation, interaction with Robo receptors, and crosstalk with neurotrophin pathways—provides a comprehensive understanding of how tumors can actively remodel their neuronal microenvironment to support progression.

Furthermore, Sema4F predominantly influences axonal development; however, its interactions with immune-associated molecules such as CD72 and Tim2—albeit less robust—suggest that it may also have multifaceted roles in both neural and immune system functions (65). For instance, investigations into breast cancer utilizing the N-methyl-N-nitrosourea (MNU)-induced rat model revealed an increased density of nerve fibers within tumor tissues. Significantly, sensory nerves were observed to be more abundant than parasympathetic nerves (66).

This observed distribution of nerve types, coupled with a disproportionate ratio between sympathetic and parasympathetic nerves, is intricately associated with the onset and advancement of cancer (67). Veena et al. found that highly metastatic mouse mammary tumors gained more innervation than tumors with less metastasis (68). This enhanced innervation was driven by the expression of the axon guidance molecule SLIT2 in the tumor vasculature (69). SLIT2, a secreted ligand, canonically signals through its Roundabout (Robo) family receptors to mediate chemorepulsive cues during neuronal development and vascular patterning (70). Its expression in the tumor microenvironment repels or guides specific nerve subtypes, facilitating tumor innervation. Recent studies reveal a more complex interaction network, showing that SLIT2 can also bind to Neuropilin-1 (NP1), a receptor typically associated with class 3 semaphorins and vascular endothelial growth factor (VEGF). This binding is competitive and occurs at the b1 domain of NP1, which may antagonize the binding of other ligands like SEMA3A, thereby modulating axonal guidance and endothelial cell responses in a context-dependent manner (71). The engagement of SLIT2 with NP1 can influence downstream signaling cascades independent of Robo receptors. This interaction has been shown to activate pathways involving Src family kinases and focal adhesion kinase (FAK), leading to alterations in cytoskeletal dynamics and cell adhesion—processes critical for both neurite extension and tumor cell invasion (72). Breast cancer cells induced spontaneous calcium activity in sensory neurons, triggered the release of neuropeptide substance P (SP), and promoted breast tumor growth, invasion, and metastasis. In a similar vein, various malignancies, including head and neck squamous cell carcinoma, pancreatic ductal adenocarcinoma, cervical cancer, and basal cell carcinoma, exhibit reliance on sensory nerves. Conversely, prostate cancer cells promote the proliferation of sympathetic nerves, establishing a reciprocal dynamic that fosters tumor growth via adrenergic receptor activation, ultimately contributing to chemoresistance (73).

The results underscore that axonogenesis linked to cancer, particularly within sensory and sympathetic nerve pathways, represents a notable emerging characteristic of malignancies. Within the tumor microenvironment (TME), the interplay between neoplastic cells and the neuronal framework is essential, not only for tumor advancement but also for modulating immune responses and affecting therapeutic efficacy. Additional investigations are warranted to elucidate the mechanisms through which cancer stimulates axonogenesis, especially concerning parasympathetic nerves, and to assess the prospective advantages of targeting this pathway in oncological therapies (15).

## 4 The impact of neural innervation on tumor biology

### 4.1 Neural regulation of tumor angiogenesis

Angiogenesis, defined as the development of new blood vessels from pre-existing ones, plays a critical role in tumor growth and progression (74). A pivotal element in this process is neural innervation, particularly from adrenergic nerves, which modulate angiogenesis through the release of norepinephrine (75). The interaction of norepinephrine with β-adrenergic receptors located on endothelial cells stimulates the formation of new vascular structures (76, 77). The anatomical proximity and parallel arrangement of nerves and blood vessels, especially small arteries and capillaries, are supported by shared axon guidance molecules, underscoring their complex interrelationship.

In the realm of prostate cancer, adrenergic norepinephrine has emerged as a vital contributor to the proliferation of endothelial cells and tumor expansion via β-adrenergic receptor signaling pathways (78). Evidence suggests that the deletion of the ADRB2 gene, responsible for encoding the β2-adrenergic receptor, impedes angiogenesis by enhancing oxidative phosphorylation in endothelial cells. Notably, the simultaneous knockout of both ADRB2 and COX10, a crucial component in the assembly of cytochrome c oxidase, mitigates the metabolic alterations typically induced by ADRB2 deletion, thereby decelerating prostate cancer progression. Furthermore, norepinephrine’s activation of ADRB2 triggers the cAMP-PKA signaling cascade in endothelial cells, indicating that targeting cAMP may represent a viable strategy to inhibit tumor-associated angiogenesis (79). The reliance of endothelial cells on glycolysis for directed migration during angiogenesis further underscores its biological significance (80). In cases of high-grade prostate intraepithelial neoplasia, researchers have noted an increased co-localization of neuronal fibers (TH+) and endothelial cells (CD31+), suggesting a potential correlation between reduced distances between neurons and endothelial cells and heightened malignancy. Importantly, during the initial 18 days of tumor development, Adrb2 knockout mice exhibit no significant alterations in early tumor growth, vascular permeability, or hypoxic conditions, thereby illustrating the intricate timing of these regulatory processes.

### 4.2 Neural modulation of lymphangiogenesis in tumors

Lymphatic vessels, which are innervated by sympathetic nerves, play a pivotal role in lymphangiogenesis and remodeling, thereby facilitating the dissemination of cancer cells (81). The lymphatic system is essential for immune surveillance and the maintenance of homeostasis, as it generally facilitates the drainage of interstitial fluid and cells to lymph nodes for immune assessment (82, 83).

In the context of cancer, this system can assist cancer cells in evading immune detection and act as a reservoir for prometastatic chemokines. Similar to the vascular system, lymphatic function is modulated by adrenergic and sympathetic signals. Acute activation of the sympathetic nervous system enhances the contractility of lymphatic vessels and promotes lymphocyte movement. In models of breast cancer, the release of norepinephrine induced by stress stimulates cancer cells to secrete VEGFC, which in turn fosters lymphangiogenesis, remodeling, and an increase in stromal expression of VEGFR3 (FLT4) (84). Furthermore, β-adrenergic signaling promotes the expression of COX2 in macrophages, resulting in the secretion of PGE2 and the subsequent release of VEGFC, which further facilitates metastasis (85).

These observations indicate that neurogenic catecholamines have a positive impact on lymphangiogenesis through inflammatory pathways and the VEGFC/FLT4 signaling axis. Strategies aimed at denervation have demonstrated a reduction in lymphangiogenesis and tumor aggressiveness. Concurrently, β-blockers may effectively diminish both lymph node and distant metastasis, underscoring their significant clinical relevance (86).

### 4.3 Neural influences on immunity and inflammation in tumors

The relationship between neurons and the immune system is characterized by complexity and intricacy. Neurons possess the ability to promptly identify signaling molecules that are secreted by the immune system and to respond instantaneously (87). Concurrently, immune cells are adept at detecting and responding to neurotransmitters and neuromodulatory agents. This reciprocal interaction between the nervous and immune systems plays a vital role in maintaining their standard functions and is essential in the initiation, development, and management of cancer (88).

In the context of oncology, a multifaceted interaction is observed among neurons, immune cells, and neoplastic cells. The nervous system is integral to the advancement of cancer, exerting influence on several components, including the tumor immune microenvironment, the pre-cancerous immune status, and the overall immune response of the organism towards neoplasia. Furthermore, neural activities are critical in modulating the efficacy of immunotherapeutic approaches. At the heart of this complex interplay lies the autonomic nervous system. The afferent fibers of the vagus nerve play a pivotal role in conveying peripheral immune signals to the central nervous system, while its efferent fibers are instrumental in optimizing immune responses via cholinergic mechanisms. For instance, in experimental sepsis models induced by lipopolysaccharide, vagus nerve activation markedly diminishes the secretion of pro-inflammatory cytokines, thereby facilitating enhanced regulation of the immune reaction (89). Additionally, the parasympathetic nervous system, through the release of acetylcholine, demonstrates significant anti-inflammatory properties, whereas the sympathetic nervous system contributes to this pathological paradigm by modulating immune responses, influencing immune cell behavior, and governing the inflammatory milieu.

Neural signals, particularly norepinephrine, play a crucial role in modulating the functionality of immune cells and the secretion of inflammatory cytokines through the activation of β-adrenergic receptors (90). This signaling pathway has the potential to suppress the activation of CD8+ T cells, thereby facilitating immune evasion by tumors (91). The interplay between the nervous and immune systems fine-tunes their respective functions, which has profound implications for cancer progression (92). For example, gamma-aminobutyric acid (GABA) released by B cells can inhibit the immune response in colon adenocarcinoma by activating GABAA receptors on T cells (93). In a similar vein, serotonin produced by platelets in the context of pancreatic and gastric cancers enhances the expression of programmed death-ligand 1 (PD-L1) on tumor cells, thereby diminishing the efficacy of cytotoxic T cells. These intricate interactions underscore the dynamic relationship among neurons, immune cells, and cancer cells, collectively influencing the tumor microenvironment and ultimately affecting clinical outcomes (95).

Neural signals, particularly norepinephrine, play a crucial role in modulating the functionality of immune cells and the secretion of inflammatory cytokines through the activation of β-adrenergic receptors (β-ARs) (90). The downstream pathway by which norepinephrine inhibits CD8+ T cells involves the canonical Gαs protein-coupled signaling initiated upon β-AR engagement. This leads to the activation of adenylate cyclase, a subsequent increase in intracellular cyclic AMP (cAMP) levels, and the activation of protein kinase A (PKA) (96). PKA then phosphorylates and modulates the activity of key transcription factors such as cAMP response element-binding protein (CREB). This signaling cascade results in the transcriptional alteration of genes critical for T cell effector functions, including the downregulation of interferon-gamma (IFN-γ) and granzyme B, while potentially upregulating inhibitory receptors like PD-1, thereby facilitating tumor immune evasion (91). The interplay between the nervous and immune systems fine-tunes their respective functions, which has profound implications for cancer progression (92).

Regarding the GABAergic mechanism, the statement that “GABA secreted by B cells inhibits T cells” requires clarification on receptor specifics. Functional GABAA receptors are expressed on the surface of CD4+ and CD8+ T cells and are composed of subunits such as α1, β2, and γ2 (93, 94). The binding of GABA to these ionotropic receptors triggers an influx of chloride ions (Cl^-^), leading to membrane hyperpolarization. This hyperpolarization can dampen T cell receptor (TCR) signaling by elevating the activation threshold and interfering with the early calcium (Ca²^+^) flux that is essential for NFAT activation and downstream cytokine production (97). For example, gamma-aminobutyric acid (GABA) released by B cells can inhibit the immune response in colon adenocarcinoma by activating these GABAA receptors on T cells and suppressing their proliferative and cytotoxic capacities (93). In a similar vein, serotonin produced by platelets in the context of pancreatic and gastric cancers enhances the expression of programmed death-ligand 1 (PD-L1) on tumor cells, thereby diminishing the efficacy of cytotoxic T cells. These intricate interactions underscore the dynamic relationship among neurons, immune cells, and cancer cells, collectively influencing the tumor microenvironment and ultimately affecting clinical outcomes (95).

The immune landscape of the central nervous system (CNS) is uniquely structured, featuring an internal lymphatic system that facilitates intricate communication with immune cell populations residing within the cranial bone marrow of the skull. This interaction fosters diverse neuroimmune dynamics in the brain’s peripheral areas. Such dynamics can substantially affect the efficacy of immunotherapeutic strategies, giving rise to distinctive neuro-immune-tumor interactions both within the CNS and in peripheral tissues. Furthermore, the nervous system is pivotal not only in modulating immune responses but also in encoding these responses into the brain’s immune memory, which has the potential to alter subsequent immune activities. This phenomenon highlights the profound regulatory capacity of the nervous system over immune functions. For instance, immune responses initiated in the gastrointestinal tract can be encoded by neurons located in the insular cortex, allowing for their reactivation in response to specific immune stimuli, thus effectively reinstating the original immune response pattern (98). This scenario exemplifies the extensive influence of neuron-activity-dependent mechanisms within the realm of cancer biology.

In conclusion, neural innervation exerts profound effects on tumor biology, modulating angiogenesis, lymphangiogenesis, and immune-inflammatory responses. Understanding these intricate mechanisms offers novel therapeutic avenues for targeting tumor progression and metastasis.

## 5 Therapeutic exploitation of the nervous innervation in cancer management

### 5.1 Inhibiting tumor growth via neural modulation

The complex interplay between neurons and tumor cells, commonly referred to as tumor-nerve synapses, presents a promising strategy for cancer therapy (99). These synaptic connections leverage neural signaling mechanisms to activate pathways that promote tumor proliferation (100). Notably, in the context of high-grade gliomas and retinoblastomas, adjacent neurons and oligodendrocyte precursor cells secrete a protein identified as soluble neuroligin-3 (NLGN3) (101). This observation suggests the potential for repurposing existing neuromodulatory agents as therapeutic options for cancer treatment (102). Retrospective analyses and limited clinical trials have indicated that β-adrenoceptor antagonists may enhance cancer prognoses, reinforcing the concept of utilizing established medications for novel applications, particularly in brain tumors where numerous neuromodulators can readily penetrate the blood-brain barrier (103). For instance, a prospective study in patients with pancreatic ductal adenocarcinoma found that the use of non-selective β-blockers was associated with significantly longer overall survival, suggesting a direct impact on disease progression (104). Furthermore, a pilot clinical trial investigating the preoperative use of the β-blocker propranolol in breast cancer patients demonstrated that this treatment reduced biomarkers of tumor invasion and metastasis, such as E-cadherin and S100A4 expression, within the surgical specimens (105). A significant obstacle in clinical settings is the general lack of tumor specificity exhibited by these agents, which often results in undesirable side effects at therapeutic doses.

### 5.2 Reshaping the immuno-oncologic microenvironment

Immunocheckpoint inhibitors have become a cornerstone in the treatment of various malignancies, highlighting the necessity to comprehend the interactions between the nervous system and immune cells within tissues and lymphatic structures. This interplay suggests that the concurrent administration of neuroactive medications with immunocheckpoint inhibitors, cytotoxic therapies, or cancer vaccines may enhance the immunological milieu and amplify antitumor responses. This concept is being actively tested in clinical settings. For instance, a phase I clinical trial investigating the combination of the non-selective beta-blocker propranolol with the anti-PD-1 immunotherapy pembrolizumab in patients with metastatic melanoma demonstrated that the combination was safe and associated with a higher overall response rate compared to historical controls receiving pembrolizumab alone (106). Furthermore, in patients with melanoma, the use of nonselective beta-blockers has been correlated with a reduced risk of disease recurrence and improved overall survival among those receiving immunotherapy (107). The presence of adrenergic and sensory nerve fibers in lymph nodes plays a pivotal role in modulating T-cell trafficking, indicating that neural signaling significantly impacts both innate immune responses and those augmented by checkpoint inhibitors (108). Furthermore, the diminished presence of CD8+ T cells in ‘cold’ tumors—characterized by a poor response to checkpoint blockade—implies that the integration of beta-blockers with modalities such as radiotherapy or immunotherapy may yield advantageous outcomes (109).

### 5.3 Targeting cancer metastasis via neural pathways

The involvement of the nervous system in the process of cancer metastasis is gaining recognition as both a conduit and a facilitator for the dissemination of cancer through the circulatory and lymphatic systems (110). The interplay between vascular endothelial growth factor (VEGF), metastasis, angiogenesis, and vascular permeability is well established in the literature (111).

Nevertheless, the integrated regulation of angiogenesis and neurogenesis underscores the significance of neurotrophic factors, such as nerve growth factor (NGF) and brain-derived neurotrophic factor (BDNF), in the aberrant formation of blood vessels (112). While preclinical data is compelling, direct clinical evidence targeting these pathways in metastasis is emerging. For instance, a phase II trial of tanezumab, a monoclonal anti-NGF antibody, in patients with bone metastases from prostate cancer reported significant reductions in pain scores, a key driver of the neuro-metastatic cascade; however, its direct impact on metastatic burden requires further investigation in oncology-specific trials (113). Research utilizing murine models of breast cancer has demonstrated that the administration of anti-NGF antibodies can significantly diminish the metastatic spread to the liver and reduce the density of microvessels (114). Furthermore, the nervous system is pivotal in the context of brain metastasis, as breast cancer cells leverage pre-existing neural pathways to establish structures referred to as pseudo-tripartite synapses (33). These synapses are characterized by the release of glutamate, which is instrumental in promoting tumor proliferation.

### 5.4 Managing neuropathic pain by targeting sensory nerves

Intense discomfort at the locations of both primary and metastatic tumors, frequently linked with neural infiltration, highlights the significant involvement of neurotrophic factors and neuromodulators in pain associated with cancer (115). For instance, nerve growth factor (NGF) stimulates sensory fibers located in the dorsal root ganglia, which plays a crucial role in the pain experienced by individuals with pancreatic cancer (116). While the primary aim of managing cancer-related pain is to alleviate symptoms, addressing neuropathic pain pathways may also influence tumor advancement. This consideration is critical, as the signaling proteins involved in neuropathic pain regulation can simultaneously facilitate tumor growth, rendering these pathways attractive targets for therapeutic interventions (117). A pertinent example of this strategy is the application of NGF-targeting monoclonal antibodies, such as tanezumab, which are currently undergoing Phase III clinical trials for the treatment of painful bone metastases, potentially providing the dual advantages of alleviating pain while controlling tumor growth. Furthermore, resiniferatoxin, a potent analog of capsaicin, selectively ablates sensory nerve fibers in murine models, leading to pain reduction and deceleration of tumor progression (118). This compound is presently in Phase I trials and exhibits potential for the management of severe or treatment-resistant pain in patients with advanced malignancies (119).

Intense discomfort at the locations of both primary and metastatic tumors, frequently linked with neural infiltration, highlights the significant involvement of neurotrophic factors and neuromodulators in pain associated with cancer (115). Emerging research reveals that tumor-derived signaling can directly activate nociceptive neurons to drive pain. For instance, in HPV-positive head and neck cancers, small extracellular vesicles secreted by tumor cells are taken up by TRPV1+ nociceptors, triggering neuronal hyperexcitability and pain behaviors, identifying a novel mechanism of cancer-induced nociception (120). Furthermore, nerve growth factor (NGF) stimulates sensory fibers located in the dorsal root ganglia, which plays a crucial role in the pain experienced by individuals with pancreatic cancer (116). While the primary aim of managing cancer-related pain is to alleviate symptoms, addressing these neuropathic pain pathways may also influence tumor advancement. This consideration is critical, as the signaling proteins involved in neuropathic pain regulation can simultaneously facilitate tumor growth, rendering these pathways attractive targets for therapeutic interventions (117). A pertinent example of this strategy is the application of NGF-targeting monoclonal antibodies, such as tanezumab, which are currently undergoing Phase III clinical trials for the treatment of painful bone metastases, potentially providing the dual advantages of alleviating pain while controlling tumor growth. Furthermore, resiniferatoxin, a potent analog of capsaicin that targets TRPV1, selectively ablates sensory nerve fibers in murine models, leading to pain reduction and deceleration of tumor progression (118). This compound is presently in Phase I trials and exhibits potential for the management of severe or treatment-resistant pain in patients with advanced malignancies (119).

In summary, the application of neural mechanisms in cancer therapy encompasses a variety of approaches. These approaches are designed to inhibit tumor proliferation, bolster the immune response towards neoplasms, hinder the dissemination of cancer, and alleviate pain associated with the condition. Such initiatives underscore the intricate interplay between the nervous system and cancer, while simultaneously opening avenues for innovative treatment options that merge oncology with neuroscience. Continued research in this domain is anticipated to enhance our comprehension of the interactions between the nervous system and cancer. This understanding is poised to herald a new phase of precision medicine that leverages the body’s neural networks in the fight against cancer.

### 5.5 Future perspectives and challenges in tumor neurobiology

Recent developments in the field of tumor neurobiology have revealed that complex interactions between neoplastic cells and the nervous system extend beyond tumor innervation to critically influence therapeutic outcomes. Such findings pave the way for innovative therapeutic approaches (121). For instance, cancer-induced nerve injury has been identified as a key mechanism that promotes local immunosuppression and resistance to anti-PD-1 immunotherapy, highlighting the nervous system’s role in modulating treatment response (122). This underscores the potential of targeting neural signaling pathways, such as the metabotropic glutamate receptor 4 (mGluR4), to remodel the tumor microenvironment and enhance anti-tumor immunity (123). Ongoing investigations in this domain are anticipated to enhance our comprehension of the fundamental mechanisms involved, particularly the molecular processes that facilitate communication between tumors and adjacent neural structures. This research will necessitate an examination of the intricate signaling pathways that modify nerve architecture within the tumor microenvironment, which may contribute to the advancement of cancer (124). A critical aspect of this crosstalk is how neural infiltration mediates tumor metabolic reprogramming, which subsequently impairs the efficacy of immunotherapy in cancers like non-small cell lung cancer (125). Furthermore, the identification of novel biomarkers and therapeutic targets linked to tumor-associated nerves presents significant opportunities for the formulation of targeted therapies (126). Promisingly, targeted pharmacological intervention in nerve-cancer crosstalk has been shown to enhance the efficacy of standard chemotherapy in pancreatic cancer, validating this axis as a viable therapeutic target (127, 128). By focusing on these neural elements, it may be possible to disrupt the signaling pathways that promote tumor proliferation and dissemination, thereby impeding cancer progression and metastasis (129).The progression of this discipline encounters numerous technical hurdles. A primary obstacle lies in the necessity for accurate diagnostic instruments capable of visualizing and quantifying neural infiltration within neoplasms (130). Although existing imaging modalities provide useful insights, they frequently exhibit deficiencies in sensitivity and specificity. Such limitations hinder a comprehensive evaluation of tumor neurobiology (131, 131). Furthermore, the formulation of personalized therapeutic approaches that cater to the distinct neurobiological characteristics of each tumor poses a considerable challenge. To overcome this issue, it is crucial to amalgamate advanced genomic, proteomic, and imaging technologies. This synthesis will facilitate a more precise characterization of the interactions between tumors and nerves at the molecular level.

In order to address the complexities inherent in tumor neurobiology and propel the discipline forward, it is imperative to foster interdisciplinary collaboration. The interactions between tumors and nerves are intricate and necessitate the integration of knowledge from a diverse array of specialists, including those in oncology, neuroscience, immunology, bioengineering, and computational biology (43). By uniting researchers from these varied fields, we can harness their distinct perspectives and expertise to tackle the multifaceted challenges associated with tumor-nerve interactions. Such collaborative efforts are poised to facilitate the creation of groundbreaking diagnostic methodologies. Furthermore, they will improve the precision of treatment targeting and expedite the application of fundamental scientific findings into tangible clinical practices.

## 6 Conclusion

The evolving field of tumor neurobiology has established that neural innervation and neuro-immune crosstalk are critical drivers of cancer progression. This review highlights mechanisms, such as adrenergic and glutamatergic signaling, that shape an immunosuppressive microenvironment. Clinical translation has successfully demonstrated the feasibility and preliminary efficacy of repurposing drugs like β-blockers alongside immunotherapy. However, significant challenges remain, including a lack of tumor specificity, potential on-target side effects, and an absence of validated predictive biomarkers to guide patient selection. For future impact, researchers must prioritize delineating precise mechanistic targets and discovering robust biomarkers. Clinicians should advocate for and participate in larger, randomized trials designed to confirm efficacy and establish a precision medicine approach for neuromodulatory combinations. Overcoming these hurdles through interdisciplinary collaboration is essential to translate these discoveries into improved patient outcomes.
